# A worked example of "best fit" framework synthesis: A systematic review of views concerning the taking of some potential chemopreventive agents

**DOI:** 10.1186/1471-2288-11-29

**Published:** 2011-03-16

**Authors:** Christopher Carroll, Andrew Booth, Katy Cooper

**Affiliations:** 1 Health Economics and Decision Science (HEDS), School of Health and Related Research (ScHARR), University of Sheffield, Sheffield, UK

## Abstract

**Background:**

A variety of different approaches to the synthesis of qualitative data are advocated in the literature. The aim of this paper is to describe the application of a pragmatic method of qualitative evidence synthesis and the lessons learned from adopting this "best fit" framework synthesis approach.

**Methods:**

An evaluation of framework synthesis as an approach to the qualitative systematic review of evidence exploring the views of adults to the taking of potential agents within the context of the primary prevention of colorectal cancer.

**Results:**

Twenty papers from North America, Australia, the UK and Europe met the criteria for inclusion. Fourteen themes were identified *a priori *from a related, existing conceptual model identified in the literature, which were then used to code the extracted data. Further analysis resulted in the generation of a more sophisticated model with additional themes. The synthesis required a combination of secondary framework and thematic analysis approaches and was conducted within a health technology assessment timeframe.

**Conclusion:**

The novel and pragmatic "best fit" approach to framework synthesis developed and described here was found to be fit for purpose. Future research should seek to test further this approach to qualitative data synthesis.

## Background

While the potential limitations of qualitative data synthesis are frequently articulated, so is the utility of conducting such analysis[1]. Framework synthesis is one of several methodologies currently being developed for synthesising qualitative data [2]. This type of synthesis is based on framework analysis[3] and "offers a highly structured approach to organising and analysing data (e.g. indexing using numerical codes, rearranging data into charts etc.)" [2]. It involves the preliminary identification of *a priori *themes against which to map data from included studies. In contrast to such methods as meta-ethnography[4], framework synthesis is primarily a deductive approach. As such it carries certain pragmatic advantages which might prove beneficial within the constraints of a health technology assessment where effectiveness review, economic evaluation and qualitative evidence synthesis are conducted together within tight time constraints. Thus a framework may not simply be an instrument for analysis but may also represent a scaffold against which findings from the different components of an assessment may be brought together and organised. Limited numbers of published examples of "framework synthesis" exist, among which the most prominent have been produced by the same team at the Institute of Education, University of London[5-7]. The present synthesis therefore represents an early worked example of this approach, the only one originating from outside of the team who developed the method, and offers an opportunity for further methodological advances. It is also the first to explore the strengths and limitations of a pragmatic "best fit" approach using an existing conceptual model as a starting point to identify *a priori *themes.

This qualitative evidence synthesis was originally designed to complement a systematic review and economic evaluation on the prevention of colorectal cancer by reviewing evidence relating both to the attitudes of adults concerning the taking of named chemopreventive agents and factors that may inform the related, perceived risk-benefit balance[8]. The agents of interest were non-steroidal anti-inflammatory drugs (NSAIDs, including aspirin), vitamins, minerals, folic acid or folate, selenium, calcium and dietary supplements generally. No previous evidence synthesis was identified regarding people's views about taking these agents, especially for primary prevention of colorectal cancer. The effectiveness of any agent is moderated by levels of compliance with the proposed regimes. For those contemplating taking such agents, for example to protect against cancer, the decision-making process can be seen as complex, due to the uncertainty of the "trade-off" between efficacy of the agent, i.e. the likelihood of getting the cancer, and its possible long-term side effects [9]. It has also been pointed out that people may find it difficult to incorporate a regular pattern of chemoprevention into the demands of day-to-day life. On the other hand research points to the successful use of low-dose aspirin in reducing the risk of heart attack and stroke[10].

The aim of the current paper is to summarise key results of this synthesis of qualitative studies within the context of describing the application of a "best fit" method, and to consider the lessons learned from adopting such an approach to framework synthesis.

## Methods

### Search methods

The aim of the qualitative evidence synthesis was to examine people's attitudes towards the taking of agents or supplements that may be used in the primary prevention of colorectal cancer, i.e. NSAIDs (including aspirin), vitamins, minerals, folic acid or folate, selenium, calcium and dietary supplements generally. The synthesis included studies that focused on exploring the views, beliefs or attitudes of people who took any of these agents for any purpose. A systematic search to identify relevant studies was performed by an information specialist following piloting of appropriate search strategies. The search combined terms describing the agents of interest (NSAIDs, aspirin, vitamins, etc.) with a published, validated filter for identifying qualitative studies, together with the medical subject heading "qualitative research"[11]. The full search strategy is available in the **Appendix**. Databases searched for published and unpublished material included MEDLINE, PreMEDLINE, CINAHL, EMBASE, AMED, ASSIA, IBSS, PsycINFO, Science Citation Index, and Social Science Citation Index, and the HMIC and King's Fund databases. Studies were limited to those in English published from 2003 onwards to capture contemporary views and attitudes. Searches were undertaken in June 2008. Given the problems with identifying social science or qualitative literature through systematic searching of electronic databases alone[12,13], the reference lists of all included studies were checked for additional literature, and a "berry-picking approach" utilising supplementary, non-systematic searching[14] testing various combinations of terms was also performed by two of the authors (AB, KC). This iterative, pragmatic approach to searching aimed to identify a set of studies providing relevant information on views and attitudes towards the taking of potential chemopreventive agents.

### Study selection

To be included in the review, a study had to focus on exploring the attitudes, perceptions and beliefs of adults (any country) surrounding the taking of the agents listed above, through qualitative data from interviews or focus groups, and cross-sectional data from satisfaction surveys, i.e. unstructured and structured, but often textual data describing people's own, personal, subjective experiences, views or attitudes relating to the intervention of interest. Previous reviews have also adopted this inclusive approach to "views" studies, i.e. including qualitative data describing people's attitudes and beliefs from satisfaction surveys as well as more traditional qualitative study designs[6,15]. The authors each screened a third of the citations for relevance (based on the inclusion criteria) and references for potential inclusion were discussed within the team. Disagreements or uncertain inclusions were resolved by discussion or by retrieval of the full paper to make a definitive judgment. Full papers of all potentially relevant citations were screened using the same process. Data from the included studies were extracted by two of the authors (CC, KC) using a review-specific form developed following piloting on one included paper.

### "Best fit" approach to framework synthesis

**Figure 1:**
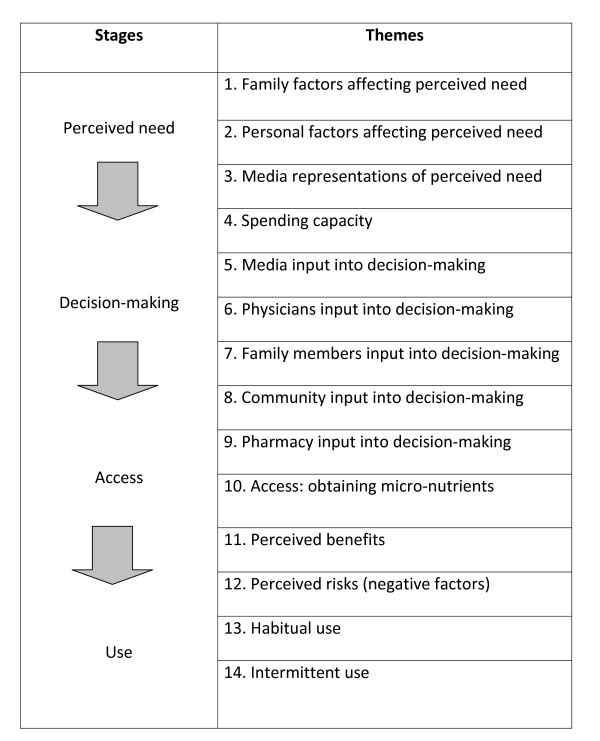
***A priori *themes reflecting people's views about taking potential chemopreventive agents, derived from Huffman 2002**[16].

The authors chose the framework synthesis approach because a published model was identified from the literature that conceptualised attitudes of adult women to the taking of vitamins and minerals[16]. The approach therefore was augmentative and deductive (building on this existing model or framework), rather than grounded or inductive (starting with a completely blank sheet). The model identified did not entirely match the topic under study, but it was a "best-fit" and provided a relevant pre-existing framework and themes against which to map and code the data from the studies identified for this review. A list of themes was derived from this model (see Figure 1) and provided the *a priori *framework of themes against which to code the data extracted from the included studies.

Data for analysis consisted either of *verbatim *quotations from study participants or findings reported by authors that were clearly supported by study data, for example, 'four of the five interviewees reported that the views of family and friends affected their decision-making' or '75% of respondents said that they were concerned about side effects of NSAIDs'. These data were extracted from the "Results" sections of included studies only, as it was felt that the Discussion and Conclusion sections would not present any new data, only additional interpretation or contextualisation of a study's 'findings'. Two of the authors (CC, KC) each extracted data from half of the included studies. Where any relevant data from the included studies did not translate into any pre-existing themes, a method was required to capture these data for the analysis. The published descriptions of framework synthesis do not specify a particular method for this, so the authors applied secondary thematic analysis, an interpretive, inductive approach grounded in the data based on methods from primary research, whereby additional themes were created as needed based on the study data[17]. In this way, the existing model acted as the basis for the synthesis and could be built-upon, expanded upon, reduced or added to by these new data. Each reviewer checked and examined critically the extraction and categorisation or coding of data performed by the other. The principal aim of this process was to examine the first reviewer's categorisation of the data, i.e. either to verify the coding or to challenge it by offering an alternative.

The authors then discussed the data and resulting themes, both those from the pre-existing model and those generated by the novel, inductive thematic analysis of the extracted study data. A consensus was reached on which *a priori *themes were supported by the data, and whether new themes identified by the reviewers did actually map either to a pre-existing theme or to one another (c.p. reciprocal translation[2]). The result was a finalised list of themes. The primary reviewer (CC) then offered an interpretation of the relationships between the themes based in part on the relationships as they were represented in the original model (see Figure 1), and also based on the data itself, which suggested, for example, that "the media" inputted into the central procedural themes of both perceived need and decision-making. The new model was then critically considered by all reviewers. A revised conceptual model was therefore developed building on the earlier, identified model, to describe and explain people's views around the taking of potential chemopreventive agents.

### Consideration of study quality

Published descriptions of framework synthesis typically exclude studies of lower quality. However this was not the approach used in this case, representing a further innovative deviation from the published method[2]. All studies that satisfied the relevance criteria were included because there is an increasingly strong case for not excluding qualitative data studies from evidence synthesis based on quality assessment[1,18,19]. Studies were assessed using key quality criteria derived from relevant critical appraisal checklists for qualitative studies[20] and other systematic reviews of people's views[1,2]. These elements also appear in recent guidance from the Cochrane Qualitative Research Methods Group[21]. The assessment consisted of querying whether the following are clearly and adequately described in the publication: the question and study design; how the participants were recruited or selected; and the methods of data collection and analysis used (See Additional file 1). The "better-reported" studies provided details on two or more criteria, whereas the "inadequately-reported" studies clearly described no more than one. The decision only to focus on these four elements, and what was reported or clearly described by the included studies, was taken for two reasons. Firstly, these elements of the study were potentially more easily judged and apprehended than others, as they were either described or not. Secondly, it has been pointed-out previously that any appraisal checklist is only assessing what has been reported in a publication[22]. The focus therefore was on the reporting of basic methods and not potentially subjective judgements regarding studies' validity or reliability[18].

While it is acknowledged that there is always uncertainty concerning how well or poorly a study has been conducted, if authors clearly describe their approach and sampling, and data collection and analysis methods, then this potentially lends greater robustness to the study's findings. This is because any inherent "risk of bias" may be better determined than if this information was absent, regardless of the study's findings. This does not preclude the possibility that an "inadequately-reported" study has actually been well-conducted, but it does form a reasonable basis for making a quality assessment. This relatively small number of easily-defined criteria can also be seen to apply to qualitative studies universally and may be more practical than checklists with much larger numbers of questions, especially as these have been found to generate low inter-rater reliability scores among otherwise experienced qualitative systematic reviewers[18]. This was one of the first practical attempts to utilise assessment criteria based specifically and exclusively on the description or reporting of a study's method and sampling strategies, and methods of data collection and analysis. No study was excluded on the basis of the adequacy of its reported processes, but the assessment aimed to explore quality of reporting as an explanation for differences in the results of otherwise similar studies, and to consider its impact on the internal validity of the review[23]. A sensitivity analysis would be performed in the event of the inclusion of "inadequately-reported" studies.

## Results

### Quantity and quality of included studies

The literature search identified 1,805 unique citations, 15 of which satisfied the inclusion criteria. Five further studies were identified by the "berry picking" approach described above[8]. In total, twenty studies were included. No study failed to describe clearly at least two of the following: the question and study design, and the methods of sampling, data collection or analysis. Study quality, in terms of how well or how poorly studies were described, was therefore not a potential moderator of the findings; a sensitivity analysis was not performed.

### Data synthesis and development of model

**Figure 2:**
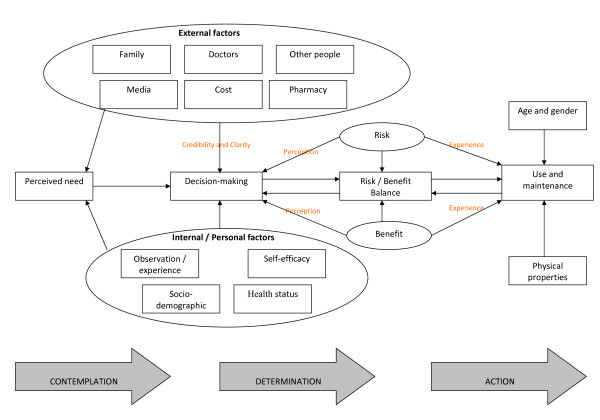
**Conceptual model to describe views and experiences of adults concerning the taking of potential chemoprevention agents**.

A combination of coding against pre-existing themes and the generation of and assignment of data to new, agreed themes, generated the model presented in Figure 2. A full description of the evidence supporting this model is published elsewhere[8]. The model describes the processes involved in an individual's decision about whether or not to take possible chemopreventive agents. The process runs from the first stages of perceived need, on the left, through the decision-making process itself, to final non-use or use, and maintenance of use, on the right. External agents, such as health professionals and family members, and internal factors, such as a person's own experience or health, were all found to impact both on an individual's perceived need for an agent or supplement, as well as their subsequent decision about whether or not to take it.

### Usefulness of the preliminary conceptual framework in assigning data to themes

Since the source of the preliminary framework was a single published model, the manner in which new themes built-on, developed and altered this preliminary conceptual framework is quite transparent. In this review, this may be assessed in part by comparing Figure 1 with Figure 2. The principal procedural elements of the preliminary model also held true for this sample of studies and their population, i.e. the transition through the stages of perceived need, decision-making, risk versus benefit and use or non-use. These elements also reflect the three key stages of Contemplation, Determination and Action in Prochaska and Velicer's model (1997) of the development of health behaviours, which was later found to be relevant[24]. The *a priori *identification of these key constructs therefore enabled the rapid coding of study data from this review against these tested and highly relevant components of health behaviour decision-making. The preliminary framework also provided "themes" that informed the "perceived need" and "decision-making" stages of the model (see numbers 1-9 in Figure 1). Once clear definitions had been applied to each of these themes, the study data were coded rapidly against them. Very little study data were coded against the themes of "Spending capacity" (or "Costs") and "Access: obtaining the agent", which may reflect differences in the cultural context of the preliminary conceptual model (a low-income country in South America) compared with the studies included in the review (principally UK, Europe and North America). However, relatively more substantial amounts of data were coded against the remaining themes.

### Extension of the preliminary conceptual framework to generate the final model

Despite these helpful overlaps, which permitted rapid and reliable coding of much data from the included studies, the preliminary model lacked sufficient depth or complexity to explain all the data in the included studies. As with the preliminary model, some factors influenced both need and decision-making. For example, the influences of family and the media were present at both of these stages, but the categorisation of these factors was re-specified in the new model. Family, media, physicians, other people and pharmacy were all designated in the new model as external factors having input into perceived need and decision-making. The "personal factors" theme from the original model was re-specified as "internal or personal factors" to include an individual's own observations or experience, their health and socio-economic status, age and gender, and their sense of self-efficacy. All of these characteristics were found in the included studies to affect perceived need and decision-making. It was felt that the pre-existing theme of "personal factors" alone was insufficient to illustrate the complexity of factors at play. The role of age, gender and the physical properties of agents were new factors identified by the synthesis affecting the *a priori *theme of use, which were absent from the original conceptual model.

Relationships between the themes were not well-developed in the preliminary model. The synthesis found that not only did family, physicians and others affect decision making, but also that this relationship was moderated by the credibility of the source and the clarity of the information being given. Perceived risks and benefits were key pre-existing themes shaping use, but the moderating role of personal experience was an additional element identified by the synthesis for the new model. Furthermore the risk/benefit balance theme was also found to have an ongoing, potentially recursive influence on decision-making and agent use. Indeed, unlike the existing models, which appear to be exclusively linear, the model that resulted from this synthesis was potentially more recursive: the decision-making stage might still be revisited on the basis of side-effects ("risks") experienced at the stage of use. This new model can therefore be seen not only to validate, but also to build upon, extend and contextualise existing, relevant published models. The *a priori *boxes of Contemplation, i.e. perceived need; Determination, i.e. decision-making; and Action, i.e. use and maintenance, have been opened to reveal the complexities of the factors therein, their relationships and moderators.

## Discussion

The model generated by the framework synthesis describes the processes involved in an individual's decision about whether to initiate and keep taking potential chemopreventive agents. External agents, such as health professionals and family members, and internal factors, such as a person's own experience or health status, combine to impact on an individual's perceived need for an agent or supplement, and their subsequent decision about whether or not to take it. Decision-making was strongly influenced by perceived risks and benefits associated with an agent or supplement. Firstly, perceived risks and benefits directly influence an individual's decision to take an agent. Secondly, they may inform a personal assessment of the trade-off between risk and benefit, thus affecting the decision-making process. It has been reported elsewhere that decision-making regarding agents for chemoprevention or symptom management may be affected both by health status, for example, a cancer diagnosis[25,26], and by people's perceived need for an agent and perceived risks associated with that agent[27-29]. The model generated by this review highlights the complex influences at work in this decision-making process.

This review applied a form of framework synthesis to analyse the data, based on a single "best fit" model identified in the literature. This approach differs from other published versions of framework synthesis in which the *a priori *framework was developed from a range of sources, including familiarisation with and consultation around the published background literature, both theoretical and empirical, and personal experiences[5,6]. The approach taken here is of potential value for systematic reviewers as it does not require such extensive literature review, consultations or topic expertise to develop an *a priori *framework before embarking on the review itself. This may be of particular value when undertaking a synthesis of qualitative evidence within the limited timeframes of a health technology assessment, for example. Projects such as Health Technology Assessments, produced in multidisciplinary centres with contractual obligations, with a six-month or one-year span, and which also involve reviews of effectiveness, cost-effectiveness, mathematical modelling and, in some cases, qualitative evidence synthesis, often present challenges in relation to timeliness and the availability and expertise of members of research teams[30]. In this particular case study, the qualitative evidence synthesis was conducted after the effectiveness synthesis, which required the qualitative synthesis to be fairly quick within the project's required timeframe. However, a temporal dependency between the two types of synthesis will not always exist, and so a more in-depth qualitative approach may be possible for some projects. However, if a framework of related, relevant concepts already exists, then the approach used here permits a far more rapid identification of the *a priori *framework; it also permits more rapid and structured coding and synthesis of data from the review's included studies than grounded-theory techniques. In this way, where existing theories or models exist, they can be tested against the evidence for the review's own particular criteria and evidence. This approach is therefore potentially more pragmatic than other forms of qualitative data synthesis. The identification and use of a model that was overtly "best fit", and therefore carried shared acknowledgment within the team that it was contingent on emerging data also empowered the reviewers to resist the inclination to "slot" study findings into a generic framework. This potentially enabled individual team members to privilege context-specific insights that emerged from this review over the generic observations already present within the pre-existing model. Furthermore it provided a mechanism for flagging up and explicitly communicating divergent findings or themes within the review team. The resultant synthetic product is expressed as an enhanced model recording each key dimension identified; the nature of the concepts under study; and associations between themes and tensions between them[6].

The method is however dependent on the identification of an appropriate existing conceptual model. The review team sought to identify such a model by combining a sensitive string of search terms (e.g. model$ OR framework$ OR theoretical OR theory OR concept OR conceptual) with terms representing the health-related behaviour of interest. This approach was employed firstly on a bibliographic database (PubMed MEDLINE) but was found to be limited by poor coverage of theoretical aspects in published abstracts. A more productive approach proved to be using Google Scholar with the same string of search terms, and certainly the potential for this approach to be used with other collections of full-text documents remains to be further explored. This strategy was conceived as iterative and purposive: it required search strategies that aimed to maximise the likelihood of retrieving a model of pragmatic utility to the project; the aim was not the systematic identification of all such models.

Furthermore the approach used for this particular case study was predicated on the review team's belief that the key criterion of the appropriateness of such a model most likely related to the health-related behaviour of interest, i.e. attitudes to the long-term taking of particular dietary supplements or similar agents. The population and the agents themselves may be less critical in such cases, although the closer the fit to the population and intervention of interest, the better. This is why we describe it as a "best-fit" approach. In this case study, young women and vitamins or micro-nutrients formed a sub-set of the populations and agents of interest. The conceptual model therefore had limited external validity but was still externally valid.

Some issues were encountered when piloting this "best fit" framework synthesis method. When initially seeking to code the extracted data from the included studies using the themes derived from the relevant model, the two reviewers were not always coding the same data against the same themes. It therefore became apparent that each of the *a priori *themes had to be clearly defined in order to facilitate the coding process. The subsequent provision of clear consensual definitions not only enhanced the reliability of the coding, but also strengthened the rigour of the synthesis. It should be recognised, however, that while consensus between reviewers strengthens internal validity this does not necessarily ensure congruence with the original meanings intended by the author of the framework (external validity). In this sense a form of "reciprocal translation" is taking place but via use of a conceptually rich "index paper" (many-to-one), rather than across all included studies (many-to-many), as intended by the originators of meta-ethnography[31]. Such considerations have been neither identified nor articulated in previous studies.

It further became apparent that additional analysis was needed to interpret and analyse data which could not be reliably assigned to any of the pre-existing, *a priori *themes, or, in the case of "personal factors", for which the pre-existing theme was inadequate. In this sense the usefulness of a particular framework is not only determined by "conceptual fit" but also by pragmatic concerns of what proportion of the study data can be accommodated within it. Further thematic analysis of data from the included studies was therefore required. This was completed by the first author using standard thematic analysis techniques, and the results examined critically by the other two reviewers. The resulting, agreed new themes were then incorporated with the pre-existing themes into a new conceptual model that captured the data and reflected a possible network of relationships between those data-driven themes. The existing published descriptions of the framework synthesis method do not detail particular techniques for analysing data that are not captured by the preliminary framework, how any such new themes are to be incorporated into the final model, or how the relationships between these themes may be expressed.

Finally, this review did not exclude studies on the basis of quality, thereby deviating from one element of the published description of framework synthesis[2]. The internal validity of a review depends in part on the quality of included studies and the reliability of their findings. Currently there is much debate and little consensus around the feasibility and usefulness of quality assessments of qualitative studies in evidence synthesis[18]. Some techniques, such as meta-ethnography[4], and the previously published form of framework synthesis, actively exclude studies on the basis of the quality assessment. The quality assessment for this review focused on reporting of study design, sampling strategies and methods used for data collection and analysis. These items were the most frequently reported and easily apprehended elements of study design. They thus offered a reasonable route for identification of potential risk of bias. All twenty included studies were assessed as being of similar, generally satisfactory "quality", so, from this perspective, study quality did not provide a potential explanation for any differences in findings. The issue of the inclusion or exclusion of studies for this type of synthesis, based on their assessed quality, therefore remains unresolved based on this case study.

Methodologically the authors found this "best fit" approach to framework synthesis, as developed and tested in this review, to be a useful, fairly rapid and reliable and, above all, pragmatic method of synthesising qualitative data. This "best fit" approach to synthesis was therefore found to work well overall, particularly within the role previously identified as an existing strength, namely for testing existing potentially generalisable theories and models within a specific context. However, such a "best fit" approach would benefit from further testing and refinement.

## Limitations

This is a single case study evaluating the approach described; additional studies testing this approach to qualitative evidence synthesis need to be undertaken. Also, as an approach, it is only viable if an appropriate model already exists in the literature. The other published models for framework synthesis circumvent this problem as the *a priori *framework is generated by the research team itself. It is also the case that an apparently appropriate *a priori *model may be found only to accommodate a small proportion of the data from a review's included studies. In such a case, secondary thematic analysis would form the principal approach to synthesis, thus reducing the major potential pragmatic benefits of the best-fit approach described in this paper. Reviewers must therefore exercise careful consideration of the potential external validity of existing models based on the behaviour and population of interest.

## Conclusion

This "best fit" method of framework synthesis utilised current methodological developments within qualitative data synthesis for systematic review and the production of accompanying conceptual models and frameworks. The case study was a systematic review of adults' views about taking various potential chemopreventive agents. The "best fit" framework synthesis offered a means to reinforce, critique and develop an existing published model, conceived for a different but relevant population. Being able to start from *a priori *themes, rather than generating theory grounded in data, produced a relatively rapid process when compared to more interpretative forms of synthesis. However this "best fit" method still requires analysis of data that are not captured by the preliminary model. The authors suggest that this "best fit" approach occupies a pragmatic middle ground between grounded theory-type and framework based syntheses and acknowledge the need for further evaluation.

## Competing interests

The authors declare that they have no competing interests.

## Authors' contributions

CC and AB conceived the study; CC designed the study; CC, KC and AB extracted the data and appraised included studies; CC, KC, and AB analysed and interpreted the data. CC drafted the paper and KC and AB undertook critical revision of important content of the manuscript. All authors approved the final version of the manuscript.

## Appendix

Database: CINAHL - Cumulative Index to Nursing & Allied Health Literature

Search Strategy:

1 vitamin$.tw.

2 mineral$.tw.

3 folate$.tw.

4 selenium.tw.

5 calcium.tw.

6 exp Dietary Supplements/

7 Dietary Supplementation/

8 dietary supplement$.tw.

9 non-steroidal$.tw.

10 non steroidal$.tw.

11 nonsteroidal$.tw.

12 NSAID$.tw.

13 antiinflammator$.tw.

14 anti-inflammator$.tw.

15 anti inflammator$.tw.

16 aspirin$.tw.

17 or/1-16

18 interview$.tw.

19 experience$.tw.

20 qualitative$.tw.

21 exp Qualitative Studies/

22 or/18-21

23 17 and 22

24 limit 23 to yr="2003 - 2008"

## Pre-publication history

The pre-publication history for this paper can be accessed here:

http://www.biomedcentral.com/1471-2288/11/29/prepub

## Supplementary Material

Additional file 1 **The question and study design; how the participants were recruited or selected; and the methods of data collection and analysis used**. Click here for file
