# Supplementary material for: A worked example of "best fit" framework synthesis: A systematic review of views concerning the taking of some potential chemopreventive agents
Source: BMC Med Res Methodol. 2011 Mar 16;11:29. doi: 10.1186/1471-2288-11-29 (PMC3068987; doi:10.1186/1471-2288-11-29)
Supplement: Additional file 1 — The question and study design; how the participants were recruited or selected; and the methods of data collection and analysis used. [file 1471-2288-11-29-S1.DOC]

Additional file

| **Question** | **Definition and assessment: Yes / No / Unclear** |
| --- | --- |
| The question and study design | Yes **if** **it states, eg. “a case study approach was used because . . .”, “interviews were used because . . .**”  No **if paper** **does not specify question and study design** |
| The selection of participants | Yes **if** **paper describes selection explicitly as eg. purposive, convenience, theoretical** **etc.**  No **if just details of participants are given** |
| Methods of data collection | Yes **if** **details of data collection method are given eg. piloting; topic guides for interviews; number of items in a survey; use of open or closed items; validation; etc.**  No **if** **just states “focus group” or “questionnaire”** |
| Methods of analysis | Yes **if** **details of analysis are given, eg. transcription, form of analysis (with reference), etc.**  No **if** **just states “content analysis” or data were “analysed”** |
